# Knockdown of VEGFB/VEGFR1 Signaling Promotes White Adipose Tissue Browning and Skeletal Muscle Development

**DOI:** 10.3390/ijms23147524

**Published:** 2022-07-07

**Authors:** Mingfa Ling, Xumin Lai, Lulu Quan, Fan Li, Limin Lang, Yiming Fu, Shengchun Feng, Xin Yi, Canjun Zhu, Ping Gao, Xiaotong Zhu, Lina Wang, Gang Shu, Qingyan Jiang, Songbo Wang

**Affiliations:** 1 Guangdong Provincial Key Laboratory of Animal Nutrition Control, College of Animal Science, South China Agricultural University, Guangzhou 510642, China; lingmingfa1989@163.com (M.L.); 13600482294@163.com (X.L.); quanlulu1998@163.com (L.Q.); kyanzed@163.com (F.L.); langlanglimin@163.com (L.L.); 20202025006@stu.scau.edu.cn (Y.F.); fsc15816996874@sina.com (S.F.); yixin19981019@sina.com (X.Y.); canjunzhu@scau.edu.cn (C.Z.); gaoping@scau.edu.cn (P.G.); xtzhu@scau.edu.cn (X.Z.); wanglina@scau.edu.cn (L.W.); shugang@scau.edu.cn (G.S.); qyjiang@scau.edu.cn (Q.J.); 2 National Engineering Research Center for the Breeding Swine Industry, South China Agricultural University, Guangzhou 510642, China

**Keywords:** VEGFB, VEGFR1, skeletal muscle, proliferation, differentiation, iWAT browning

## Abstract

It has been demonstrated that vascular endothelial growth factor B (VEGFB) and vascular endothelial growth factor receptor 1 (VEGFR1) play a vital role in regulating vascular biological function. However, the role of VEGFB and VEGFR1 in regulating fat deposition and skeletal muscle growth remains unclear. Therefore, this study was conducted to investigate the effects of VEGFB and VEGFR1 on fat deposition and skeletal muscle growth in mice. Our results showed that knockdown of VEGFB decreased body weight and iWAT index, stimulated the browning of mice iWAT with increased expression of UCP1, decreased the diameters of adipocytes, and elevated energy expenditure. In contrast, knockdown of VEGFB increased gastrocnemius (GAS) muscle index with increased proliferation of GAS muscle by expression of PCNA and Cyclin D1. Meanwhile, knockdown of endothelial VEGFR1 induced the browning of iWAT with increased expression of UCP1 and decreased diameters of adipocytes. By contrast, knockdown of endothelial VEGFR1 inhibited GAS muscle differentiation with decreased expression of MyoD. In conclusion, these results suggested that the loss of VEGFB/VEGFR1 signaling is associated with enhanced browning of inguinal white adipose tissue and skeletal muscle development. These results provided new insights into the regulation of skeletal muscle growth and regeneration, as well as fat deposition, suggesting the potential application of VEGFB/VEGFR1 as an intervention for the restriction of muscle diseases and obesity and related metabolic disorders.

## 1. Introduction

Adipose tissue and skeletal muscle are the most components of an adult animal’s body weight, which are highly complex and heterogeneous tissues serving a multitude of functions. The basic functions of the adipose tissues and skeletal musculature are required for daily life activities. Moreover, adipose tissue and skeletal muscle mainly contribute to carcass and meat quality in agricultural animal production. However, loss of function of adipose tissue and skeletal muscle can affect functional capacity and increase the risk of many diseases, such as muscle atrophy [1], obesity [2], and diabetes mellitus [3]. Thus, regulation and balancing of fat deposition and skeletal muscle development are pivotal to both a healthy life of human and product quality of animals.

White adipose tissue (WAT), such as inguinal white adipose tissue (iWAT), displays high physiological plasticity. The process of adipogenesis and the browning of white adipose tissue contribute significantly to fat deposition and metabolism of adipose tissue [4]. Many key transcription factors including peroxisome proliferator-activated receptor γ (PPARγ) and CCAAT/enhancer-binding proteins (C/EBPs), especially C/EBPα, as well as signal transducers and activators of transcription (STATs) are necessary to promote preadipocyte differentiation into mature adipocytes [5,6]. Uncoupling protein 1 (UCP1) was thought to be a key marker of the browning of white adipose tissue, which was originally termed thermogenin owing to its role in non-shivering thermogenesis, resides within the mitochondria, plays crucial roles in energy expenditure, increases physical activity, and improves metabolic abnormalities in mammals [6]. Moreover, one of the phenotypes of WAT browning is the decreased diameters of adipocytes in WAT. In addition, a subset of potential benefits can be obtained from the browning of white adipose tissue. Indeed, the browning of white adipose tissues has been demonstrated to play a role in the lipid metabolism of many diseases such as obesity [7], type 2 diabetes [8], and cardiovascular diseases [9].

Skeletal muscle growth and development mainly depends on the proliferation and differentiation of satellite cells in adult mammalian skeletal muscle. The satellite cells, which reside along the host muscle fiber, undergo several rounds of proliferation to increase the myogenic pool needed for muscle growth or tissue repair [10]. The satellite cell proliferation is regulated by cell cycle regulators such as Cyclin D1, Cyclin E, and cyclin-dependent kinases (CDKs) [11,12]. Following proliferation, satellite cells start to differentiate into new fibers when mature muscle is damaged or muscle growth is needed. Many transcription factors in the generation and/or regeneration of skeletal muscle have been discovered. The most classic and known myogenic regulatory factors (MRFs) are myogenic factor 5 (Myf5), myogenic determining factor (MyoD), myogenin (MyoG), and muscle-specific regulatory factor 4 (MRF4), playing essential roles in the process of adult myogenesis [13]. In addition, the myosin heavy chain (MyHC), the myotube-specific structural protein, can be regulated by these myogenic regulatory factors such as MyoD and MyoG [14]. Therefore, it is necessary to evaluate the state of proliferation and differentiation of skeletal muscle by detecting these proliferative and differentiation markers.

There are numerous factors playing a regulatory role in the process of fat deposition and metabolism of adipose tissue and skeletal muscle growth, such as growth factors [15] and nutrients [16,17]. It has been demonstrated that vascular endothelial growth factors and receptors are involved in the process of fat deposition and metabolism and skeletal muscle growth [18,19,20]. In addition, our previous study has demonstrated that vascular endothelial growth factor B (VEGFB), a vascular endothelial growth factor family member, promotes myoblast proliferation and differentiation [21]. VEGFB binds to vascular endothelial growth factor receptor 1 (VEGFR1) and neuropilin 1 (NRP1). VEGFR1 is widely expressed in non-endothelial cells, while the expression of VEGFR2 is relatively restricted to vascular endothelial cells [22]. It is generally believed that VEGFR2 is the functional receptor that transduces signals for angiogenesis [23]. Because VEGFB does not bind to VEGFR2 and VEGFA preferentially binds to VEGFR1 with a 10-fold higher affinity than VEGFR2, a limited number of VEGFA ligands would preferentially bind to VEGFR1 under health physiological conditions, which acts as a decoy receptor [24]. Therefore, inhibition of VEGFR1 would induce VEGFA binding to VEGFR2, which transduces angiogenic signals [25]. In addition, it has been reported that VEGFR1 mediated VEGFB in facilitating endothelial free fatty acid uptake via FATP3 and FATP4, affecting lipid metabolism of skeletal muscle and adipose tissue [19,26]. However, the biological role of VEGFB and VEGFR1 in the fat deposition and metabolism of adipose tissue and skeletal muscle development remains poorly understood.

Thus, this present study was conducted to investigate the effects of VEGFB and VEGFR1 on the fat deposition and metabolism of adipose tissue and skeletal muscle development in mice. To this end, the adipogenesis and browning of white adipose tissue and the proliferation and differentiation in skeletal muscle were assessed in VEGFB knockdown mice and endothelial VEGFR1 knockdown mice. Our data showed that knockdown of VEGFB/VEGFR1 signaling promotes white adipose tissue browning and skeletal muscle development in mice.

## 2. Results

### 2.1. Knockdown of VEGFB Decreased Body Weight and Increased Energy Expenditure of Mice

**Figure 1:**
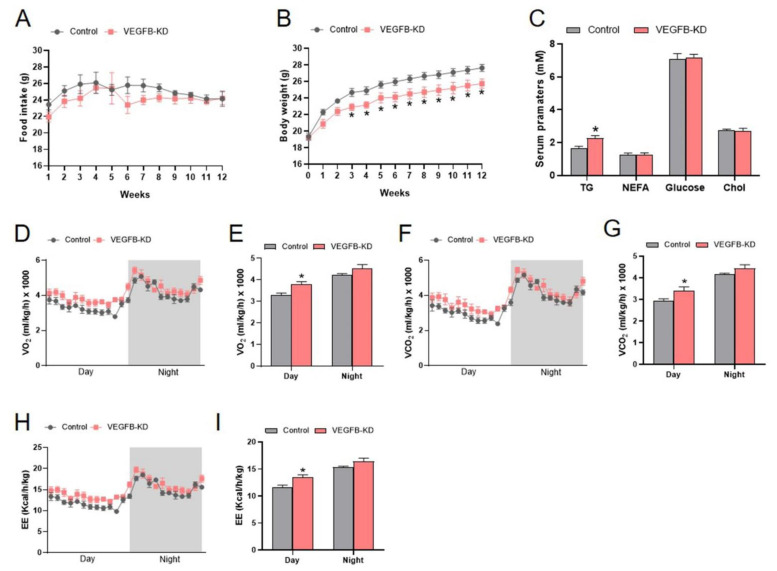
Knockdown of VEGFB decreased body weight and increased energy expenditure of mice. (**A**) The food intake of mice after AAV injection. (**B**) The body weight of mice after AAV injection. (**C**) The serum parameter concentrations of mice (TG: triglyceride, NEFA: non-esterified fatty acid, and Chol: cholesterol). (**D**,**E**) The volumes of O_2_ consumption (V_O_2__) and statistics of mean V_O_2__ of mice in a day and night cycle. (**F**,**G**) The volumes of exhaled CO_2_ (V_CO_2__) and statistics of mean V_CO_2__ of mice in a day and night cycle. (**H**,**I**) The energy expenditure (EE) and statistics of mean EE of mice in a day and night cycle. Grey areas indicate night periods. Each group contains 6 mice, values are presented as mean ± SEM, * *p* < 0.05 versus control group.

In the present study, the AAV-Cas9 combined with AAV-sgRNA targeted to VEGFB was used for VEGFB knockdown (VEGFB KD) in mice. The result showed that the knockdown of VEGFB significantly decreased the body weight of mice with no significant changes in food intake in VEGFB KD mice compared to the control group (Figure 1A,B). In addition, the knockdown of VEGFB significantly increased TG content in the plasma of mice (Figure 1C). However, there were no significant changes in insulin tolerance and glucose tolerance between the two groups (Figure S1A–D). Meanwhile, the energy metabolism of mice was measured by metabolic chambers. The data showed that the knockdown of VEGFB significantly elevated the O_2_ consumption, CO_2_ exhaled, and energy expenditure (EE) during the daytime (Figure 1D–I). Consistently, the locomotor activity tests showed that the knockdown of VEGFB tended to increase the global energy metabolism of mice (Figure S2A,B). These data suggested that the knockdown of VEGFB decreased the body weight and enhanced the energy expenditure of mice.

### 2.2. Knockdown of VEGFB Stimulated the Browning of iWAT in Mice

**Figure 2:**
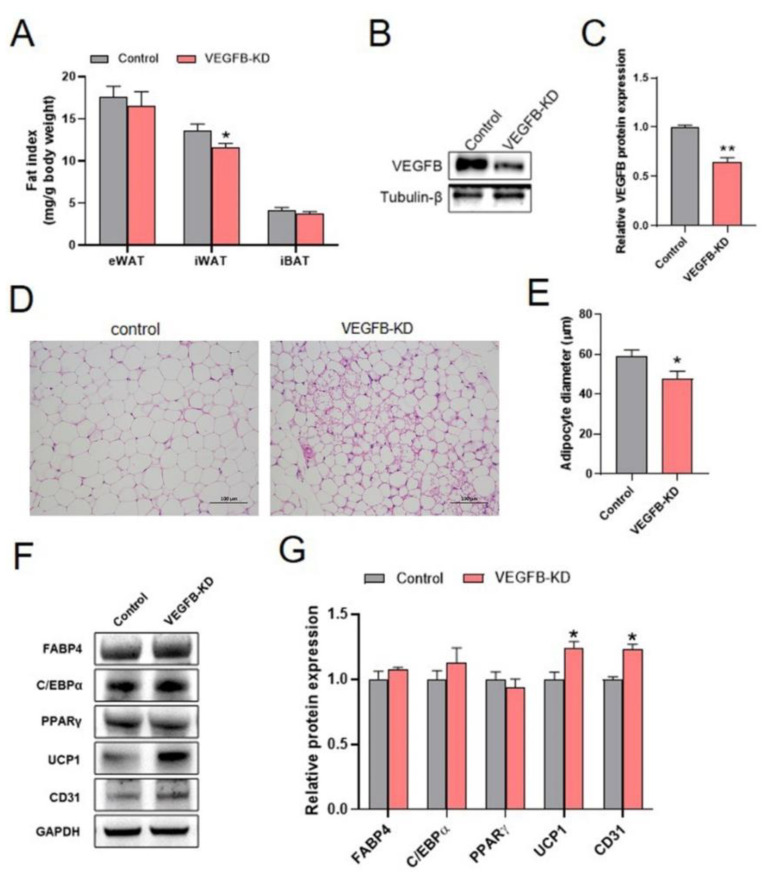
Knockdown of VEGFB decreased iWAT index and stimulated the browning of iWAT in mice. (**A**) The adipose tissue weight normalized to body weight of mice (eWAT: epididymal white adipose tissue, iWAT: inguinal white adipose tissue, and iBAT: interscapular brown adipose tissue). (**B**,**C**) WB detection of VEGFB in the iWAT of mice, the result is expressed as arbitrary unit. (**D**,**E**) Representative H&E images and average adipocytes diameters of iWAT from each group of mice. Scale bars = 100 µm. (**F**,**G**) Western blot and quantification of FABP4, C/EBPα, PPARγ, UCP1, and CD31 protein content in the iWAT. Each group contains 6 mice, values are presented as mean ± SEM, * *p* < 0.05 and ** *p* < 0.01 versus control group.

With the decreased body weight and enhanced energy metabolism of VEGFB KD mice, we evaluated the effects of knockdown of VEGFB on the fat deposition and metabolism of mice. Our results showed that the knockdown of VEGFB significantly decreased the iWAT index of mice (Figure 2A). Thus, the adipogenesis and the browning of iWAT were further accessed. To this extent, the VEGFB knockdown efficiency was validated as well. The result showed that VEGFB KD led to significant reduction in the protein expression of VEGFB in the iWAT of mice, indicating the successful knockdown of VEGFB (Figure 2B,C). Hematoxylin and eosin (H&E) staining of iWAT sections showed that the knockdown of VEGFB significantly decreased the diameter of adipocytes (Figure 2D,E). In addition, the protein expressions of UCP1 and CD31 in iWAT were significantly increased by the knockdown of VEGFB (Figure 2F,G). These data suggested that the knockdown of VEGFB induced the browning of iWAT in mice.

### 2.3. Knockdown of VEGFB Increased Gastrocnemius Muscle Index with Elevated Proliferation in Mice

**Figure 3:**
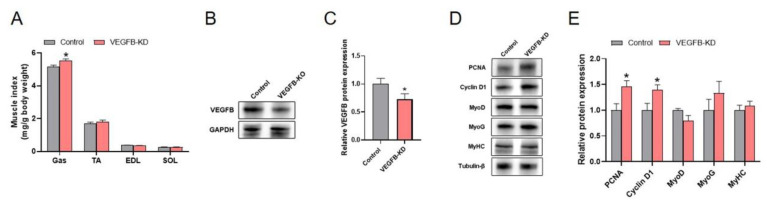
Knockdown of VEGFB increased GAS muscle index by elevated proliferation with increased expression of Cyclin D1. (**A**) The skeletal muscle weight normalized to body weight of mice (GAS: gastrocnemius, TA: tibialis anterior, SOL: soleus, and EDL: extensor digitorum longus). (**B**,**C**) WB detection of VEGFB in the GAS muscle of mice, the result is expressed as arbitrary units. (**D**,**E**) Western blot and quantification of PCNA, Cyclin D1, MyoD, MyoG, and MyHC protein content in the GAS muscle. Each group contains 6 mice, values are presented as mean ± SEM, * *p* < 0.05 versus control group.

We also investigated the effect of knockdown of VEGFB on the skeletal muscle growth. Our results showed that knockdown of VEGFB significantly increased the gastrocnemius (GAS) muscle index (Figure 3A). Herein, the VEGFB knockdown efficiency was validated by the result that VEGFB KD led to significant reduction in the protein expression of VEGFB in the GAS muscle of mice (Figure 3B,C). Based on the improvement of GAS muscle growth, the proliferation and differentiation of the GAS were accessed. The results showed that the knockdown of VEGFB increased the proliferation in the GAS muscle with elevated protein expression of PCNA and Cyclin D1 (Figure 3D,E). However, the knockdown of VEGFB had no effects on the differentiation of GAS muscle, with comparable protein expression of MyoD, MyoG, and MyHC between the two groups (Figure 3D,E). Collectively, these findings suggested that the knockdown of VEGFB promoted GAS muscle growth with increased proliferation.

### 2.4. Knockdown Effect of Endothelial VEGFR1 on the Growth and Energy Expenditure of Mice

**Figure 4:**
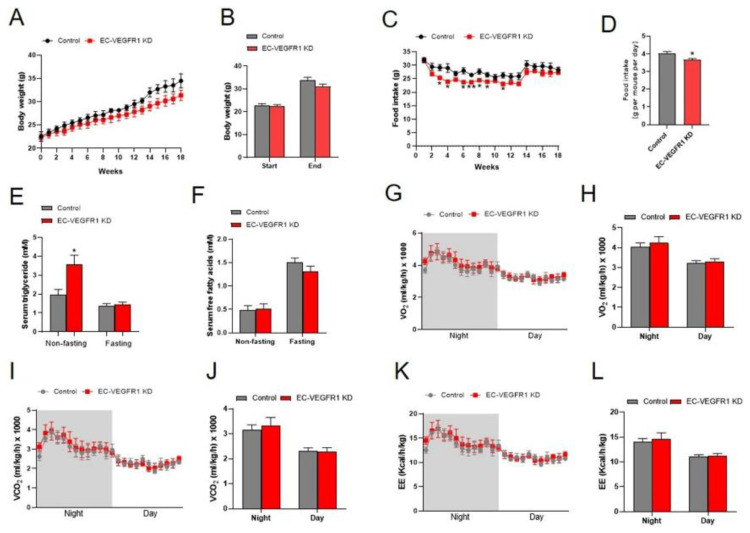
Knockdown effect of endothelial VEGFR1 on the growth and energy expenditure of mice. (**A**,**B**) Body weight after AAV injection (**A**) and the start and end body weight (B) of mice. (**C**,**D**) The weekly food intake (**C**) and average daily food intake (**D**) of mice. (**E**,**F**) The fasting and non-fasting serum triglyceride and non-esterified fatty acid of mice. (**G**,**H**) The volumes of O_2_ consumption (V_O_2__) and statistics of mean V_O_2__ of mice in a day and night cycle. (**I**,**J**) The volumes of exhaled CO_2_ (V_CO_2__) and statistics of mean V_CO_2__ of mice in a day and night cycle. (**K**,**L**) The energy expenditure (EE) and statistics of mean EE of mice in a day and night cycle. Grey areas indicate night periods. Each group has 5 mice. Data are presented as mean ± SEM.* *p* < 0.05 versus control group.

VEGFB binds to VEGFR1, and VEGFR1 is abundant and restricted expression to endothelial cells. Thus, the endothelial VEGFR1 was knocked down by the CRISPR/Cas9 system in the present study. Our results showed that the knockdown of endothelial VEGFR1 had no significant influence on the body weight of mice, whereas significantly decreased the average daily food intake (Figure 4A–D). In addition, knockdown of endothelial VEGFR1 significantly increased non-fasting TG level in the plasma of mice (Figure 4E). However, the knockdown of endothelial VEGFR1 did not change the insulin tolerance and glucose tolerance compared to the control group (Figure S3A–D). Meanwhile, the energy metabolism of mice was measured by metabolic chambers. The data showed that the knockdown of endothelial VEGFR1 had no significant influence on the O_2_ consumption, CO_2_ exhaled, and energy expenditure (EE) (Figure 4G–L). Together, these results indicated that the knockdown of endothelial VEGFR1 did not affect the body weight and the energy metabolism of mice.

### 2.5. Knockdown of Endothelial VEGFR1 Stimulated the Browning of iWAT in Mice

**Figure 5:**
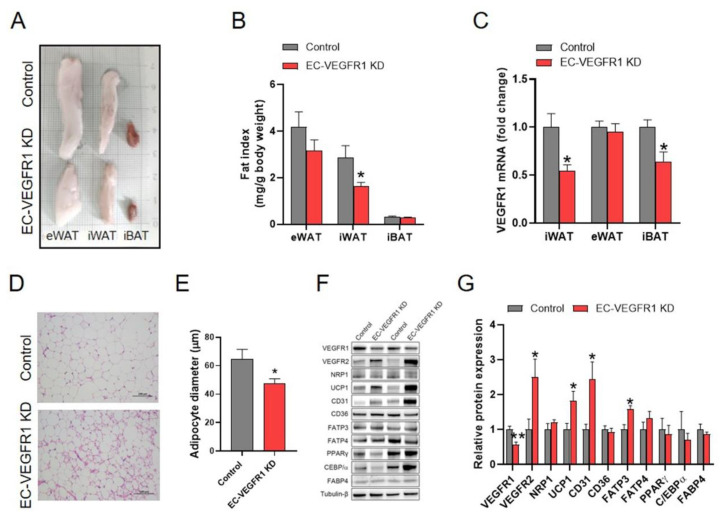
Knockdown of endothelial VEGFR1 decreased iWAT index and stimulated the browning of iWAT in mice. (**A**,**B**) Representative images of SAT and BAT obtained from each group of mice (**A**) and fat mass normalized by body weight (**B**). (**C**) The VEGFR1 mRNA expression of WAT and BAT from each group of mice. (**D**,**E**) Representative H&E images (**D**) and average adipocyte diameters (**E**) of iWAT from each group of mice. Scale bars = 100 µm. (**F**,**G**) Western blot (**F**) and quantification (**G**) of VEGFR1, VEGFR2, NRP1, UCP1, CD31, FATP3, FATP4, C/EBPα, PPARγ and FABP4 protein content in the iWAT, the results are expressed as arbitrary units. Each group contains 5 mice, values are presented as mean ± SEM, * *p* < 0.05 versus control group.

The effects of the knockdown of endothelial VEGFR1 on the fat deposition of mice were accessed. The VEGFR1 knockdown efficiency was validated by the results that the knockdown of endothelial VEGFR1 led to a significant reduction in the mRNA levels of VEGFR1 in the iWAT and iBAT, as well as the protein expression of VEGFR1 in the iWAT of mice, indicating the successful knockdown of endothelial VEGFR1 in the iWAT and iBAT (Figure 5B,C). Similar to the VEGFB knockdown mice, the knockdown of endothelial VEGFR1 significantly decreased the iWAT index of mice. Meanwhile, hematoxylin and eosin (H&E) staining of iWAT sections showed that the knockdown of endothelial VEGFR1 significantly decreased the diameters of adipocytes (Figure 5D,E). Moreover, the protein expressions of UCP1 and CD31 were significantly increased by the knockdown of endothelial VEGFR1 in iWAT (Figure 5F,G). However, the knockdown of endothelial VEGFR1 had no effects on the expression of FABP4, C/EBPα, and PPARγ, indicating no change in adipogenesis in the iWAT of mice (Figure 5F,G). Collectively, these findings suggested that the knockdown of endothelial VEGFR1 promoted the browning of iWAT in mice.

### 2.6. Knockdown of Endothelial VEGFR1 Did Not Affect GAS Muscle Index but Inhibited Its Differentiation

**Figure 6:**
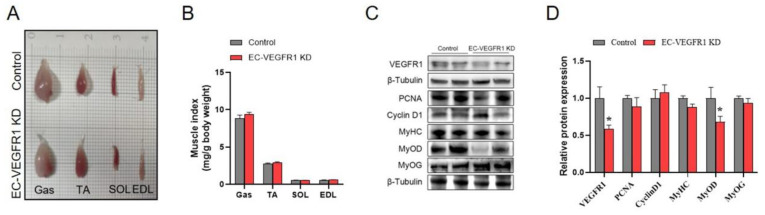
Knockdown of endothelial VEGFR1 inhibited GAS muscle differentiation of mice. (**A**,**B**) Representative images of skeletal muscle obtained from each group of mice (**A**) and muscle mass normalized by body weight (**B**). (**C**,**D**) Western blot (**C**) and quantification (**D**) of VEGFR1, PCNA, Cyclin D1, MyoD, MyoG, and MyHC protein content in the GAS muscle, the results are expressed as arbitrary units. Each group contains 5 mice, values are presented as mean ± SEM, * *p* < 0.05 versus control group.

Next, we explored the effects of endothelial VEGFR1 knockdown on the skeletal muscle growth of mice. The results showed that the knockdown of endothelial VEGFR1 had no significant influence on the skeletal muscle index (Figure 6A,B). Although the GAS muscle mass index did not significantly change as a result of the endothelial VEGFR1 knockdown, the protein expression of MyoD significantly decreased, indicating that the process of skeletal muscle differentiation had been influenced (Figure 6C,D). In contrast, the knockdown of endothelial VEGFR1 had no significant influence on the proliferation of GAS muscle with the comparable protein levels of PCNA and Cyclin D1. Herein, the successful endothelial VEGFR1 knockdown in the GAS muscle was validated by the significant reduction in the protein expression of VEGFR1 (Figure 6C,D). Together, these data suggested that the knockdown of endothelial VEGFR1 was involved in GAS muscle differentiation.

## 3. Discussion

In the present study, the effects of VEGFB and VEGFR1 on fat deposition and skeletal muscle growth was investigated. Our results demonstrated that the loss of VEGFB/VEGFR1 signaling is associated with the enhanced browning of inguinal white adipose tissue and gastrocnemius (GAS) muscle development. VEGFB plays a crucial role in regulating adipose development, fat deposition, and energy metabolism. Previous studies have reported that VEGFB knockdown mice displayed higher body weight, expansion of white adipose, and a reduction in energy consumption [27,28]. However, our present study found that the knockdown of VEGFB significantly decreased the body weight and iWAT mass index, which can be explained by the browning of iWAT with increased protein expression of UCP1, decreased diameters of adipocytes, and enhanced energy metabolism of mice. These inconsistent results may partly be due to different technical systems for the downregulation of VEGFB. Because there are great differences, we used viral vectors to interfere the knockdown of VEGFB, while VEGFB knockout mice were generated from embryos in previous studies, which resulted in the complete deletion of VEGFB compared to our technical systems [27,28]. Moreover, the partially blocked VEGFB is primarily due to the technical systems used in our present study, resulting in marginal significant differences in serum triglycerde and fat loss of iWAT. However, more importantly, the loss of VEGFB induced a reduction in VEGFR1 and the impaired VEGFB/VEGFR1 signaling in iWAT elevated the VEGF/VEGFR2 signaling, promoting angiogenesis with increased protein expression of CD31. Browning of white adipose tissue and increase in energy expenditure are usually accompanied by increased angiogenesis [29,30]. In addition, it has reported that VEGFB knockout mice have normal basal plasma levels of triglycerides and non-esterified fatty acids compared to the wild-type mice [26]. However, the knockdown of VEGFB significantly increased serum TG content in the present study. As VEGFB is a paracrine factor [26,31], coordinating endothelial-cell-mediated long-chain fatty acid uptake, the increased TG content in the plasma of VEGFB knockdown mice might be caused by the different energy demands of the surrounding tissues. Despite targeting VEGFB as a novel treatment for insulin resistance and type 2 diabetes [32], our results showed that the knockdown of VEGFB did not affect the glucose tolerance and insulin tolerance. Similarly, VEGFB ablation in pancreatic β-cells did not affect glucose homeostasis or islet lipid uptake [33]. Collectively, our results indicated that the knockdown of VEGFB was involved in the browning of iWAT.

Postnatal skeletal muscle growth and repairment largely depends on myoblast proliferation and differentiation, which can be mediated by a variety of factors, such as growth factors and nutrition [15,34]. It has been revealed that VEGFB plays a vital role in regulating different kinds of cell-type proliferation and differentiation in vivo and in vitro, including cardiomyocytes and endothelial cells [35,36]. Our previous study also demonstrated that VEGFB promotes myoblast proliferation and differentiation via VEGFR1-PI3K/Akt signaling pathway in vitro [21]. Although the functional effects of VEGFB on the fatty acid metabolism in skeletal muscle and the heart have been well demonstrated, to the best of our knowledge, the biological role of VEGFB in skeletal muscle development in vivo is currently unknown. Our present study found that the knockdown of VEGFB promoted GAS muscle growth by increasing proliferation with increased protein expression of PCNA and Cyclin D1. In contrast, many studies reported that the loss of VEGFB is associated with decreased cell proliferation and/or increased cell death in endothelial cells [37], cardiomyocytes [38], and neurones [39]. These inconsistent biological functions of VEGFB might be due to the different cell types and treatment systems. Moreover, the same cell type may display different phenotypes in vivo and in vitro, considering the cell to cell communication.

VEGFB binds to VEGFR1, activating downstream signaling and displays biological functions. VEGFR1 is abundant and restricted expression to endothelial cells. Thus, it is necessary to explore the effects of endothelial VEGFR1 on fat deposition and skeletal muscle development. To this end, the loss of VEGFR1 in mice endothelial cells was generated. Previous studies reported that the ablation of endothelial VEGFR1 augmented adipose angiogenesis and induced adipose tissue browning via VEGF/VEGFR2 signaling [19,25]. As expected, we found that the knockdown of endothelial VEGFR1 resulted in the browning of iWAT with increased protein expression of UCP1 and decreased diameters of adipocytes. Similarly, the browning of iWAT in the present study may be caused by the elevated angiogenesis with increased protein expression of VEGFR2 and CD31. Collectively, these observations suggested that the knockdown of endothelial cell-specific VEGFR1 was involved in white adipose tissue browning.

Our results showed that the knockdown of endothelial VEGFR1 had no significant influence on GAS muscle index. However, the knockdown of endothelial VEGFR1 significantly inhibited the differentiation in GAS muscle by the decrease in protein expression of MyoD. Similarly, Clément d’Audigier et al. reported that VEGFR1 inhibition of endothelial progenitors downregulates their differentiation potential in vivo and in vitro [40]. In addition, some studies indicated that endothelial cell-specific VEGFR1 deletion increased the coronary vasculature and induced cardiomyocyte hypertrophy in healthy adult mice [41] and also increased vascular density, increased satellite cell numbers, and improved functions in Duchenne muscular dystrophy mice [42,43]. Additionally, these authors explained that endothelial VEGFR1 deletion improved heart growth and repairment via VEGFR2-mediated angiogenesis. However, the effects of endothelial VEGFR1 loss on skeletal muscle angiogenesis have not been evaluated, which is a limitation of this study.

**Figure 7:**
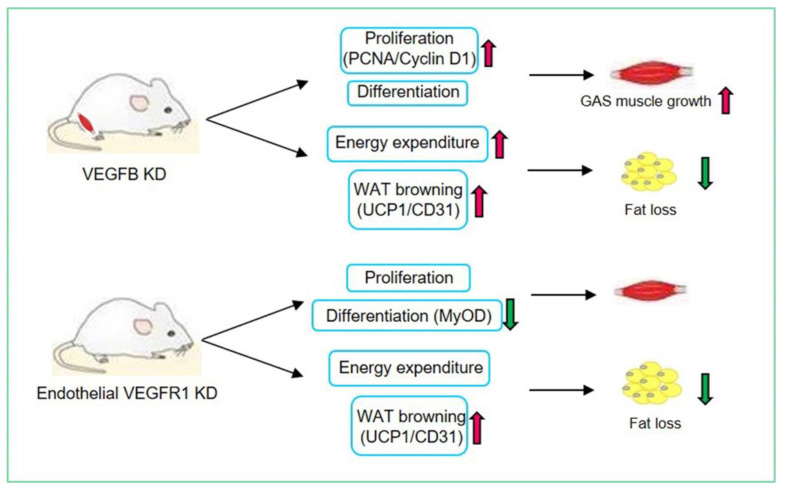
Effects of VEGFB and VEGFR1 on white adipose tissue browning and skeletal muscle growth.

In conclusion, our findings indicate that the knockdown of VEGFB/VEGFR1 signaling promotes white adipose tissue browning and skeletal muscle development as described in Figure 7. These results provide new insights into the regulation of skeletal muscle growth and regeneration and fat deposition.

## 4. Materials and Methods

### 4.1. Materials and Reagents

AAV9-CMV-spCas9-pA, AAV9-(H1-sgRNA(VEGFB)sp)×3-CAG-DIO-mCherry, AAV9-(H1-sgRNA(VEGFR1)sp)×3-CAG-DIO-mCherry, and AAV9-H1-sgRNA(NC)sp-CAG-DIO-mCherry were designed and validated by Shanghai Taitool Bioscience Co., Ltd. Antibodies against PCNA and Cyclin D1 were purchased from Zen-bioscience Company (Chengdu, Sichuan, China). Antibodies against VEGFB, VEGFR1, CD31, and MyoG were purchased from Abcam (Cambridge, MA, USA). Antibodies against VEGFR2, NRP1, and MyoD were purchased from Santa Cruz Biotechnology, Inc. (Dallas, TX, USA). Antibody against MyHC was purchased from R&D systems (Minneapolis, MN, USA). Antibodies against PPARγ, C/EBPα, and FABP4 were purchased from Cell Signaling Technology, Inc. (Danvers, MA, USA). Antibodies against UCP1, CD36, FATP3, and FATP4 were purchased from Proteintech Group, Inc. (Rosemont, IL, USA). Antibodies against β-tubulin and GAPDH were purchased from Bioworld Technology, Co, Ltd. (Nanjing, China). The goat anti-mouse HRP-conjugated secondary antibody and goat anti-rabbit HRP-conjugated secondary antibody were purchased from Bioworld Technology, Inc. (St. Louis Park, MN, USA).

### 4.2. Animals

Animal experiments were approved by the College of Animal Science, South China Agricultural University. A total of 12 three-week-old C57BL6/J male mice were obtained from the Medical Experimental Animal Center of Guangdong Province. The Rosa26-LSL-Cas9 knockin mice (Stock No: 024857) and B6; 129-Tg (Cdh5-cre) 1Spe/J (Stock No: 017968) mice were purchased from The Jackson Laboratory. All mice were housed in individual cages with a relatively stable temperature (25 ± 1 °C) and humidity (55 ± 5%) and a 12 h light/dark cycle. The mice were given ad libitum access to a standard chow diet and water.

### 4.3. Adeno-Associated Viral Vector (AAV) Injection

For the knockdown of VEGFB, 12 4-week-old C57BL6/J male mice were randomly divided into two groups according to body weight. AAV9-CMV-spCas9-pA combined with AAV9-(H1-sgRNA(VEGFB)sp)×3-CAG-DIO-mCherry was injected into the GAS muscle of both hind limbs of the mice for the knockdown of VEGFB (VEGFB-KO group). Meanwhile, AAV9-CMV-spCas9-pA combined with AAV9-H1-sgRNA(NC)sp-CAG-DIO-mCherry was injected into the GAS muscle of both hind limbs of the mice as a negative control (control group). The above-mentioned combined virus was directly injected into the skeletal muscle with three points of both hind limbs of the mice (1.39 × 10^11^ genome copies totally in each skeletal muscle). The validated sgRNA sequence for the knockdown of VEGFB is (target 1: GTCGGACTTGGTGTTGCCCAG target 2: GCGTGCATAAACGTCTATCCA target 3: GTCAGGGCGTTGACGGCGCT). After virus injection, body weight and food intake were recorded every week. The experimental operations of different groups were taken in turns at the same time, 4 weeks after virus injection. At the end of the experiment (12 weeks), the mice were sacrificed for the collection of blood, skeletal muscle, and adipose tissue samples.

For the knockdown of endothelial VEGFR1 in mice, theCas9/Cdh5-cre male mice were generated by crossbreeding the Rosa26-LSL-Cas9 knockin mice with B6;129-Tg(Cdh5-cre)1Spe/J mice. Genotypes were identified according to the genotyping protocols provided by The Jackson Laboratory. Then, at 5 weeks old, the AAV9-(H1-sgRNA(VEGFR1)sp)×3-CAG-DIO-mCherry was injected into Cas9/Cdh5-cre mice via the tail vein to knockdown the endothelial VEGFR1 (EC-VEGFR1 KD group). AAV9-H1-sgRNA(NC)sp-CAG-DIO-mCherry was injected as a negative control (control group). The validated sgRNA sequence for the knockdown of VEGFR1 is (target 1: GTACACCTGTCGCGTGAAGAG target 2: GTACAGGTGCCCGTTGACGG target 3: GTCCTCCTGGCTCACGGTCGT). Each mouse received 2.78 × 10^11^ genome copies of the virus. After virus injection, body weight and food intake were recorded every week. The experimental operations of the different groups were taken in turns, 4 weeks after virus injection. At the end of the experiment (18 weeks), the mice were sacrificed for the collection of blood, skeletal muscle, and adipose tissue samples.

### 4.4. Metabolic Measures

For metabolic studies, mice were individually housed in metabolic chambers with free access to food and water. The energy expenditure, O_2_ consumption, and CO_2_ emissions were measured by Promethion Metabolic Screening Systems (Sable systems International, North Las Vegas, NV, USA). Data were collected after 24 h of adaptation and analyzed by MetaScreen-Data Collection Software (V2.3.17) and Expedata-P Data Analysis Software (V1.9.17), respectively.

### 4.5. Locomotor Activity

The locomotor activity of each mouse was tested in a custom-made open field chamber (50 × 50 × 50 cm), and their movement was recorded for 5 min using a video camera fixed overhead during the light phase. The total traveled distance was recorded and analyzed using SuperMaze (Xinruan Corporation, Shanghai, China).

### 4.6. Blood Parameters

The concentrations of serum glucose, non-esterified fatty acid (NEFA), triglyceride (TG), and total cholesterol (Tcho) were determined by using the biochemistry kits purchased from Nanjing Jiancheng Bioengineering Institute (Nanjing, China).

### 4.7. Glucose and Insulin Tolerance Tests

For the glucose tolerance tests (GTT), mice were fasted overnight. After measuring the baseline blood glucose level via a tail nick using a glucometer, 1 g/kg of glucose was administered via intraperitoneal injection and the glucose levels were measured 0, 30, 60, 90, and 120 min after glucose injection. One week later, the same mice were used for the insulin tolerance tests (ITT). For ITT, mice, that had fasted for 6 h, were injected intraperitoneally with insulin at 1 U/kg and their blood glucose concentrations were determined 0, 30, 60, 90, and 120 min after insulin injection.

### 4.8. Hematoxylin and Eosin (H&E) Staining

The paraffin-embedded iWAT sections were stained with H&E as described before. Pictures of stained adipose tissue were captured using an EVOS XL Core Cell Imaging System (Life technologies) and adipocyte sizes were analyzed by Image-Pro Plus 6.0 (Media Cybernetics, Inc., Rockville, MD, USA).

### 4.9. Real-Time Quantitative PCR (RT-PCR) Analysis

Total RNA was extracted from adipose tissues using TRIzol reagent according to the manufacturer’s instructions and 1 µg of total RNA was reverse transcribed to cDNA by the color transcription kit (A0010CGQ, Roseville, CA, USA). Two microliters of diluted cDNA was used in each real-time PCR assay by M×3005P (Stratagene, La Jolla, CA, USA). The following PCR cycling conditions were used for each assay enzyme activation: at 95 °C for 15 s, amplification of the gene product through 40 successive cycles of 95 °C for 5 s, 60 °C for 30 s, dissolution curve of the gene at 95 °C for 15 s, 60 °C for 30 s, and 95 °C for 15 s. Relative quantification of target mRNAs was calculated and normalized to β-actin expression by the 2^−ΔΔCt^ method. The primer sequences of VEGFR1 and β-actin were: (Accession: NM_010228.4, forward: GGAGGAGTACAACACCACGG, reverse: TTGAGGAGCTTTCACCGAAC) and (Accession: NM_007393.5, forward: ACTCTTCCAGCCTTCCTTC, reverse: ATCTCCTTCTGCATCCTGTC), respectively.

### 4.10. Western Blotting Analysis

We used RIPA lysis buffer containing 1 mM PMSF to lyse iWAT and skeletal muscle. Protein concentration was determined with a BCA protein assay kit (Thermo Fisher Scientific, Waltham, MA, USA). Fifteen to twenty micrograms of total protein was used for electrophoresis and then transferred to a polyvinylidene fluoride membrane. Primary antibodies against VEGFB (1:1000), VEGFR1 (1:1000), VEGFR2 (1:1000), NRP1 (1:1000), PCNA (1:1000), Cyclin D1 (1:1000), MyHC (1:2000), MyoG (1:2000), MyoD (1:1000), PPARγ (1:1000), C/EBPα (1:1000), FABP4 (1:1000), UCP1 (1:2000), CD31 (1:1000), CD36 (1:2000), FATP3 (1:2000), and FATP4 (1:2000) were used in Western blot analysis. β-tubulin and GAPDH (1:10,000) were selected as a loading control. Protein expression levels were detected by a FluorChem fluorescent imaging system (Tanon 5200, Shanghai, China) and were analyzed using Adobe photoshop CS6 (Adobe, San Jose, CA, USA).

### 4.11. Statistical Analysis

Statistical analyses were carried out by using the SPSS 19.0 (IBM SPSS, Chicago, IL, USA). Data were presented as means ± SEM. *p*-values were calculated using a two-tailed unpaired Student’s t-test related to the indicated group. Values of *p* < 0.05 were considered statistically significant.

## Data Availability

All important data are included in the manuscript and raw data are available upon request.

## Supplementary Materials

The following are available online at https://www.mdpi.com/article/10.3390/ijms23147524/s1.
