# Supplementary material for: Knockdown of VEGFB/VEGFR1 Signaling Promotes White Adipose Tissue Browning and Skeletal Muscle Development
Source: Int J Mol Sci. 2022 Jul 7;23(14):7524. doi: 10.3390/ijms23147524 (PMC9315609; doi:10.3390/ijms23147524)
Supplement: Supplementary file 1 [file ijms-23-07524-s001.zip › ijms-1800910-supplementary.pdf]

Supplementary Materials

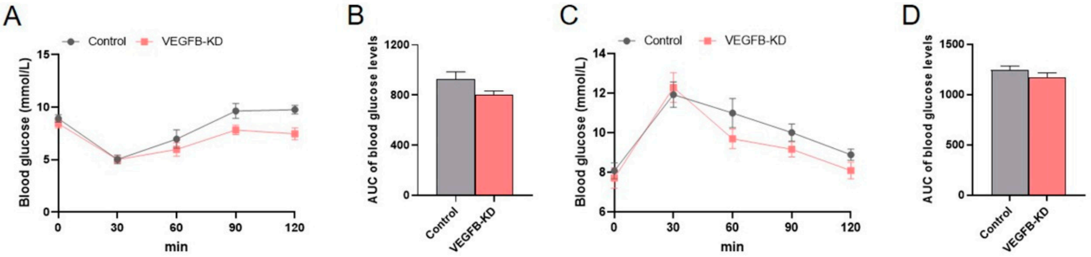

Figure S1. Effects of knockdown of VEGFB on glucose tolerance and insulin tolerance of mice. (A-B) The insulin tolerance test of mice. (C-D) The glucose tolerance test of mice. Each group contains 6 mice, values are presented as mean ± SEM, **P* < 0.05 versus control group.

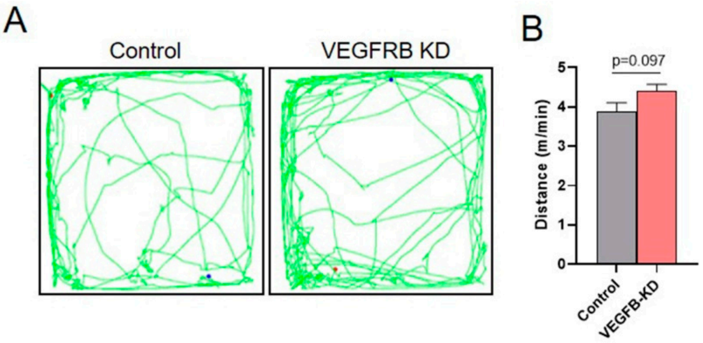

Figure S2. Effects of knockdown of VEGFB on locomotor activity of mice. (A-B) Representative trajectories and statistics of mean distances in the locomotor activity test. Each group contains 6 mice, values are presented as mean ± SEM.

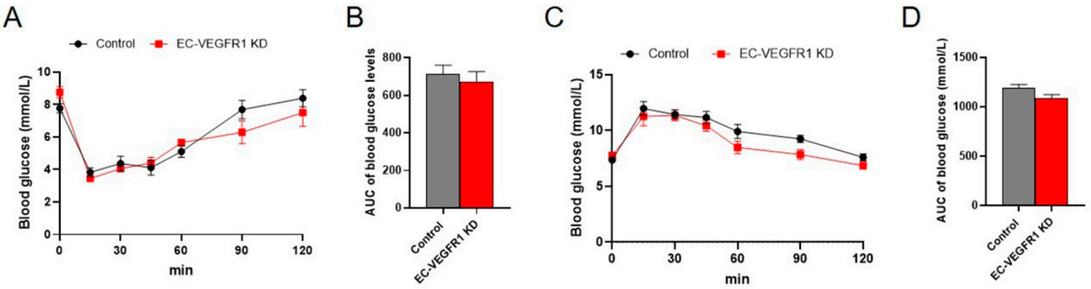

Figure S3. Effects of knockdown of endothelial VEGFR1 on glucose tolerance and insulin tolerance of mice. (A-B) The insulin tolerance test of mice. (C-D) The glucose tolerance test of mice. Each group contains 5 mice, values are presented as mean ± SEM.
